# Prognostic impact of stromal periostin expression in upper urinary tract urothelial carcinoma

**DOI:** 10.1186/s12885-022-09893-7

**Published:** 2022-07-18

**Authors:** Kosuke Miyai, Kazuki Kawamura, Keiichi Ito, Susumu Matsukuma, Hitoshi Tsuda

**Affiliations:** 1 Department of Basic Pathology, National Defense Medical College, 3-2 Namiki, Tokorozawa, Saitama 359-8513 Japan; 2 Department of Laboratory Medicine, National Defense Medical College, Tokorozawa, Saitama 359-8513 Japan; 3 Department of Urology, National Defense Medical College, Tokorozawa, Saitama 359-8513 Japan

**Keywords:** Urothelial carcinoma, Upper urinary tract, Periostin, Immunohistochemistry

## Abstract

**Background:**

Periostin is an extracellular matrix protein that has been known to be implicated in fibrillogenesis and cell migration, including cancer metastasis. Periostin overexpression in cancer cells and/or intervening stroma is usually related to tumor progression and poor patient outcomes in various human cancers; however, its role in urothelial carcinoma, especially upper urinary tract urothelial carcinomas (UTUCs), remains inconclusive.

**Methods:**

Samples from 126 consecutive cases of invasive UTUC (69 renal pelvic cancers and 57 ureteral cancers) were histologically reviewed and analyzed for periostin expression using immunohistochemistry. The intensities of immunoreactivity and the fraction of positive cancer cells and stroma (i.e., epithelial and stromal expression, respectively) were classified into four categories each (intensity, 0–3; fraction, 0–25% = 1; 26–50% = 2; 51–75% = 3; and > 75% = 4). The overall score was determined by multiplying both scores, and overall scores ≥ 6 were considered to indicate high periostin expression.

**Results:**

Among 126 UTUCs, 55 (44%; 27 renal pelvic and 28 ureteral cancers) showed high stromal periostin expression. None of the cases were considered to have high epithelial periostin expression. High stromal periostin expression was associated with non-papillary gross findings, higher pathological T category, lymphovascular invasion, concomitant carcinoma in situ, subtype histology, lymph node metastasis, positive surgical margins, high tumor budding, and high tumor-associated immune cell status. Multivariate analysis revealed that high stromal periostin expression was an independent predictor of overall survival (*p* = 0.00072, hazard ratio = 3.62), and lymphovascular invasion and high stromal periostin expression were independent predictors of cancer-specific survival (*p* = 0.032 and 0.020, hazard ratio = 2.61 and 3.07, respectively).

**Conclusions:**

Stromal periostin expression was often observed in invasive UTUCs with adverse clinicopathological factors and may be a useful predictor of patient outcomes.

## Background

Upper urinary tract urothelial carcinoma (UTUC) is defined as urothelial carcinoma located in the renal pelvis and ureter and comprises approximately 5% of all urinary tract urothelial carcinomas [1]. Radical nephroureterectomy remains the gold standard for treating non-metastatic UTUCs, whereas minimally invasive kidney-sparing procedures have been performed in selected patients with small low-grade tumors [2]. The primarily recognized postoperative prognostic factors include pathological T (pT) category, tumor grade, and lymphovascular invasion (LVI) [2–4]. However, even at the same pathological stage and tumor grade with standard treatment, patients still have significantly divergent prognoses. For example, renal pelvic cancers invading peripelvic adipose tissue or renal parenchyma (from microscopic minimal to macroscopic deep invasion) are categorized as pT3 and show highly variable clinical courses [5, 6]. We previously demonstrated that tumor budding, defined as a single cancer cell or clusters of fewer than five cancer cells at the tumor invasion front, is a possible prognostic factor independent of the stage and grade of invasive UTUCs [7]. Considering tumor invasiveness, much attention has been focused on the interaction between cancer cells and the tumor microenvironment [8, 9]; however, the molecular mechanism in UTUCs is not widely understood.

Periostin, also known as osteoblast-specific factor 2, is an extracellular matrix protein that promotes integrin-dependent cell adhesion and motility and plays a role in maintaining mechanical stress in normal tissues such as bones, teeth, and heart valves [10]. The overexpression of periostin by cancer stroma and/or neoplastic epithelium itself has been reported in various types of cancer cell lines and tissues, including breast, colon, thyroid, ovarian, prostate, and gastric cancers, and correlates with cell proliferation, invasiveness, epithelial-mesenchymal transition, metastasis, and worse patient survival outcomes [11–15]. At the molecular level, periostin activates phosphatidylinositol-3-kinase/protein kinase B (PI3K/Akt) and/or mitogen-activated protein kinase pathways by interacting with integrin receptors to promote cell adhesion, motility, and angiogenesis [13, 16]. Additionally, periostin is an inflammatory/immune factor that recruits and polarizes tumor-associated macrophages and activates T helper 2 (Th2) lymphocytes [17, 18], both of which support cancer progression and chemoresistance. However, few reports have investigated periostin expression in urothelial carcinoma of the urinary bladder, and the results remain controversial [19, 20]. Periostin expression and its role in cancer progression in UTUCs have not yet been reported.

In this study, we histologically reviewed surgically resected specimens from 126 patients with invasive UTUCs. Immunohistochemistry for periostin was performed to determine whether (1) periostin overexpression in cancer cells and/or stroma surrounding cancer is a common finding in invasive UTUCs, (2) the status of periostin expression is correlated with clinicopathological parameters, especially tumor budding status and histological tumor-associated immune cell status (TAICs), and (3) periostin overexpression has an impact on overall and cancer-specific survival.

## Methods

### Ethics arrival and consent to participate

This study was performed in accordance with the Declaration of Helsinki and approved by the Ethics Committee of the National Defense Medical College (registration number: 4007). All patients agreed to participate in this study, and written informed consent was obtained from all patients.

### Cases enrolled

Two hundred and thirteen consecutive cases with primary UTUC (117 renal pelvic tumors and 112 ureteral tumors, including concurrent tumors in the same cases described below) who had undergone radical nephroureterectomy or partial ureterectomy at the National Defense Medical College Hospital between 1999 and 2018 were included in the present study. Of these, 35 cases without invasive urothelial carcinoma (28 pTa and seven pTis), 21 cases without sufficient follow-up data, nine cases with distant metastasis at diagnosis, and 11 cases treated with neoadjuvant chemotherapy were excluded. Additionally, five patients with pT4 cancers and six patients who had simultaneously undergone total cystectomy for concurrent bladder cancer were also excluded. When concurrent renal pelvic and ureteral cancers were detected in the same patient, a tumor with a higher pT category was further investigated. After adjusting for the above exclusion criteria, there were six renal pelvic and five ureteral cancers with concomitant lower-T-category ureteral and renal pelvic cancers, respectively. Finally, 126 UTUCs (69 renal pelvic and 57 ureteral cancers) met the inclusion criteria for the present study. The extent of regional lymph node dissection was often limited (e.g., only the renal hilum for UTUC in the upper ureter). Regional lymph node dissection was performed in 43 (34%) of 126 patients with suspected enlarged lymph nodes detected during intraoperative inspection or with suspected advanced clinical stage.

### Histological evaluation

**Fig. 1:**
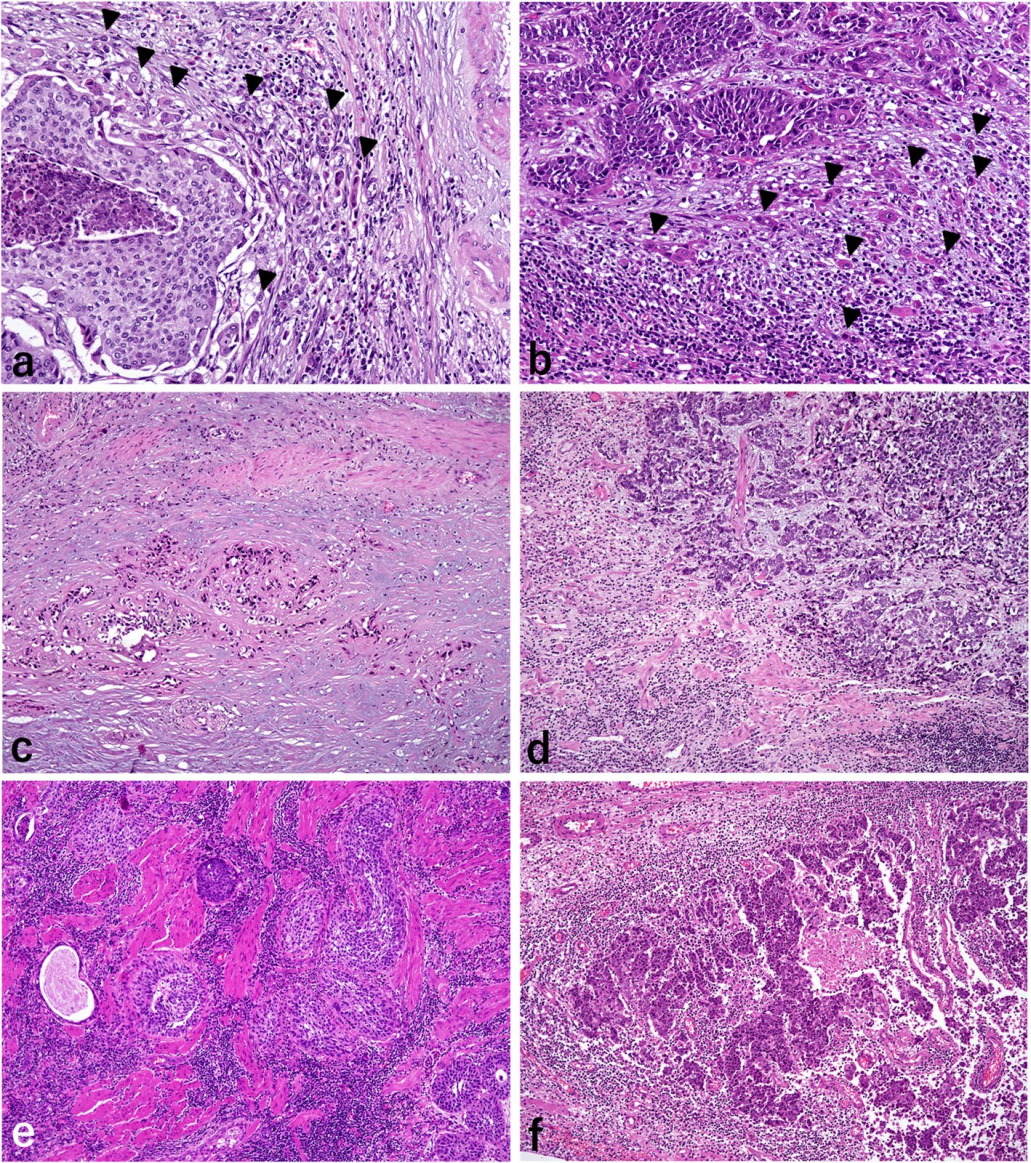
Histological findings of tumor budding and tumor-associated immune cell status (TAICs). **a** Renal pelvic cancer and **b** ureteral cancer showing evidence of tumor budding, which is defined as a single cancer cell or clusters of less than five cancer cells (arrow heads). Panels (**c**–**f**) exhibiting TAICs: **c** none, grade 0; **d** patchy infiltration, grade 1; **e** band-like infiltration, grade 2; and **f** prominent with intratumoral infiltration. Hematoxylin and eosin stain, original magnification × 200 for (**a**) and (**b**); and × 200 for (**c**), (**d**), (**e**), and (**f**)

A total of 126 UTUC cases were retrieved from the files of the Department of Laboratory Medicine at the National Defense Medical College. Two experienced surgical pathologists (KM and HT) reviewed all hematoxylin and eosin (H&E)-stained slides of the specimens to confirm the pathological findings according to the World Health Organization (WHO) criteria [21]. Tumor grades were evaluated according to the 2004/2016 WHO criteria (two categories: low and high). The pT categorization of the disease was performed according to the eighth edition of the American Joint Committee on Cancer Staging Manual [22]. Based on the International Tumor Budding Consensus Conference reporting system [23], 10 separate fields (20 × objective) along the invasive front were scanned before counting the tumor buds (cancer cells isolated or in small clusters of fewer than five cancer cells) in the single selected “hotspot.” The intensity of tumor budding in the hotspot was classified into the following three-tier system: low budding (0–4 buds), intermediate budding (5–9 buds), and high budding (10 or more buds). Histological TAICs were evaluated based on the intensity of mononuclear cells and granulocytes at the deepest interface of the carcinoma with stroma and scored as 0 (none), 1 (patchy infiltrate), 2 (band-like infiltrate), or 3 (prominent with intratumoral infiltrate) [24, 25]. For the statistical analysis, the scores were stratified as follows: low TAICs (score, 0–1) and high TAICs (score, 2–3) [24, 25]. Figure 1 shows representative cases of high budding or high TAICs.

### Immunohistochemistry

Representative blocks of the lesions were cut into 4-μm thick sections and subjected to immunohistochemistry. Deparaffinized sections were subjected to autoclave antigen retrieval using Target Retrieval Solution High pH (Dako, Glostrup, Denmark) at 120 °C for 10 min. Endogenous peroxidase was blocked for 5 min with 5% hydrogen peroxide and nonspecific binding was blocked for 10 min with 2% goat serum. The sections were then incubated with a 1:2000 dilution of rabbit polyclonal antibody against periostin (ab14041, Abcam, Cambridge, UK) at 4 °C overnight. The slides were reacted with dextran polymer reagent combined with secondary antibodies and peroxidase (Dako) for 30 min at room temperature. Specific antigen–antibody reactions were visualized with 0.2% diaminobenzidine tetrahydrochloride and hydrogen peroxide, and counterstaining was performed with Mayer’s hematoxylin. The resected colon adenocarcinoma specimen was used as a positive control. Sections without the primary antibody were used as negative controls.

**Fig. 2:**
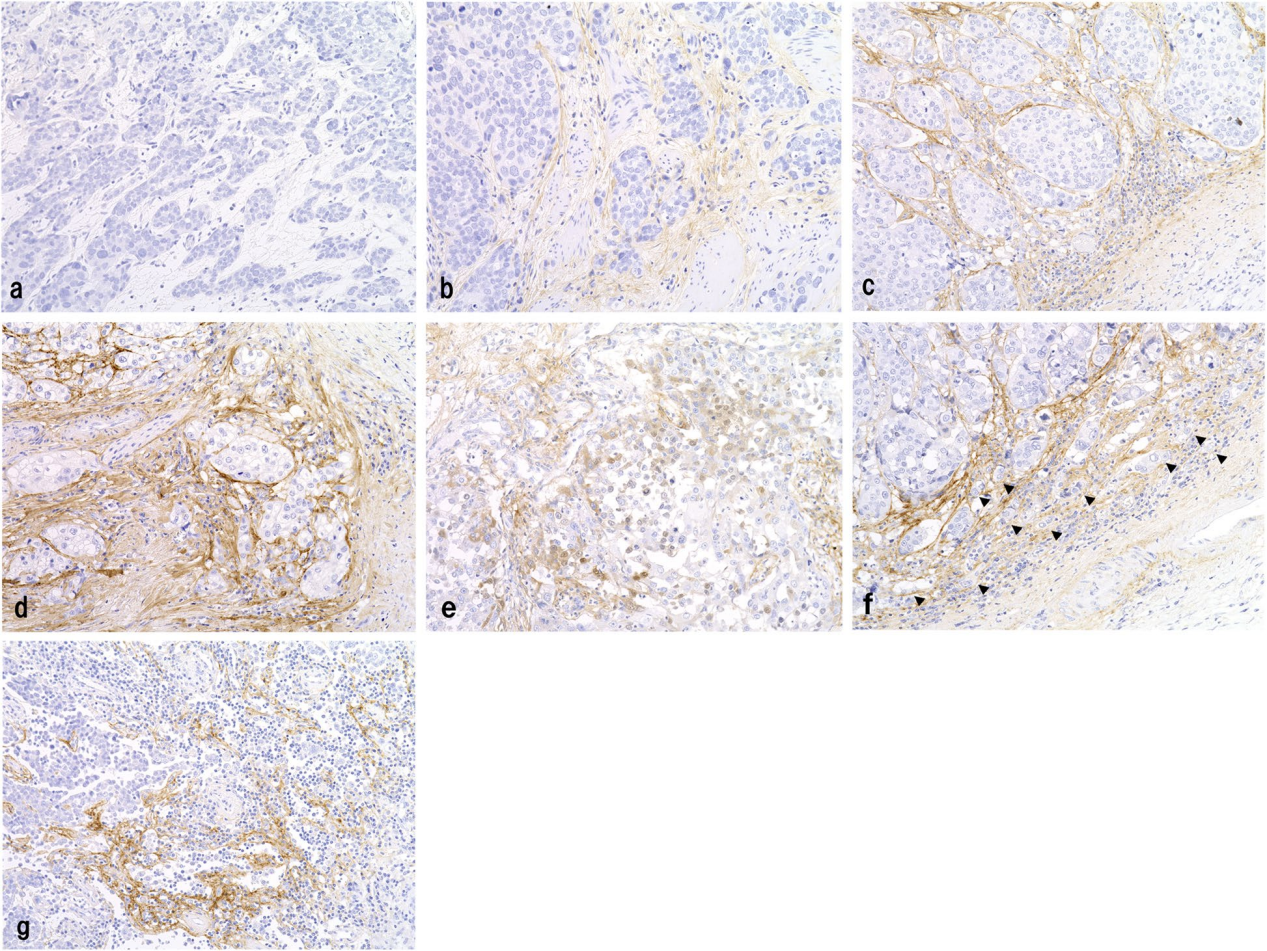
Immunohistochemical findings for periostin in upper urinary tract urothelial carcinoma. **a**–**d** Urothelial carcinomas showing (**a**) no immunoreactivity, (**b**) mild, (**c**) moderate, and (**d**) strong stromal expression of periostin. Note the absence of epithelial periostin expression in these tumors. **e** In a small subset of tumors, focal and weak epithelial expression of periostin was detected. **f** and **g** Urothelial carcinomas showing high stromal periostin expression and (**f**) high tumor budding (arrow heads)/(**g**) high tumor-associated immune status. Immunoperoxidase stain, original magnification × 200

According to the scoring system of previous reports [26, 27], periostin immunoreactivity of (1) cancer cells and (2) stroma surrounding cancer cells was separately assessed based on the predominant cytoplasmic staining intensity and the fraction of positive area. Periostin immunoreactivity intensity was classified into four categories: non-staining (score 0), weak (score 1), moderate (score 2), and strong (score 3). The fractions of positive tumor cells (in the whole tumor cells) and positive stromal cells (in the whole area of stroma surrounding the tumor cells) were estimated using a 5-titered scale (0–4% = 0; 5–24% = 1; 25–49% = 2; 50–74% = 3; and 75–100% = 4). The intensity and positive fraction scores were multiplied and the overall score (0–12) was calculated. The median value of the score (overall score = 6) was used as the cutoff point and divided into two groups: high (≥ 6) and low (< 6) periostin expression. Representative cases of periostin expression are shown in Fig. 2.

### Statistical analysis

Statistical analyses were performed using the R software (version 4.0.5, R Core Team and Foundation for Statistical Computing, Vienna, Austria). Clinicopathological parameters were compared between cases with high and low periostin expression using Fisher’s exact test or Student’s *t*-test. Overall survival was defined as the duration from the date of diagnosis to death or last follow-up, with no restriction on the cause of death. Cancer-specific survival was defined as the duration from the date of diagnosis to death due to UTUC, excluding other causes. The overall survival and cancer-specific survival rates were calculated using the Kaplan–Meier method, and comparisons were made using the log-rank test. Cox proportional hazards general linear model analysis was used to determine the impact of periostin expression and other clinicopathological variables on the overall and cancer-specific survival. Differences were considered statistically significant at *p* < 0.05.

## Results

Histological review confirmed that the tumors in all the examined cases were urothelial carcinomas. Among the 126 tumors, 55 (44%) showed high stromal periostin expression. Of these, 27 were renal pelvic cancers, and 28 were ureteral cancers. Thirty-four tumors showed divergent differentiation or subtype histology: 21 with squamous differentiation, three with glandular differentiation, three with squamous and glandular differentiation, two with trophoblastic differentiation, two with sarcomatoid subtype, one with squamous and sarcomatoid features, one with neuroendocrine and sarcomatoid features, and one with lymphoepithelioma-like subtype. Eight tumors, comprising five renal pelvic and three ureteral tumors, exhibited focal (less than 25%) weak epithelial periostin expression, and none of the examined tumors were judged to have high epithelial periostin expression (Fig. 2e).

### Relationship between stromal periostin expression and clinicopathological variables

Clinicopathological parameters and stromal periostin expression status of the examined cases are summarized in Table 1. There was no significant difference in the mean age, sex, tumor laterality, or histological grade between patients with low and high stromal periostin expression. The frequencies of tumors with non-papillary gross findings and pT category 3/4 were significantly higher in patients with high stromal periostin expression than in those with low expression (*p* = 0.00013 and *p* < 0.0001, respectively). LVI and lymph node metastasis were detected in 38 (69%) and eight (15%) cases with high stromal periostin expression and in 18 (25%) and two (3%) cases with low stromal periostin expression, respectively. A significant difference was observed in the frequency of LVI between the patients with low and high stromal periostin expression (*p* < 0.0001). With respect to the lymph node metastasis, the number of cases with pathologically positive nodes/pathologically negative nodes/no lymph node dissection was 8/17/30 and 2/16/53 in cases with high and low stromal periostin expression, respectively, with a statistically significant difference (*p* = 0.021). The frequencies of tumors with subtype histology, concomitant carcinoma in situ, and positive surgical margins were significantly higher in patients with high stromal periostin expression than in those with low expression (*p* = 0.016, *p* = 0.047, and *p* = 0.043, respectively). Of 34 tumors with subtype histology, 12 of 21 with squamous differentiation, two of three with glandular differentiation, all three with squamous and glandular differentiation, one of two with trophoblastic differentiation, one of two sarcomatoid subtype, one with squamous and sarcomatoid features, and one with neuroendocrine and sarcomatoid features showed high stromal periostin expression. Tumors with high stromal periostin expression showed more frequent high budding and high TAICs than those with low expression (tumor budding, 67% vs. 6%, *p* < 0.001; TAICs, 53% vs. 30%, *p* = 0.010) (Figs. 2f and g).

**Table 1:** Relationship between stromal periostin expression and clinicopathological parameters

Variables	Stromal periostin expression		*p* value
	Low (*n* = 71)	High (*n* = 55)	
Mean age, years^a^	72	70	0.45
Gender (male/female)^b^	52/19	43/12	0.54
Laterality (left/right)^b^	38/33	34/21	0.37
Tumor location, renal pelvis/ureter^b^	29/42	28/27	0.28
Non-papillary gross finding (%)^b^	10 (14)	25 (46)	0.00013
Pathological T category, 1/2/3/4^b^	26/18/27/0	5/6/42/2	< 0.0001
Tumor grade, low/high^b^	3/68	0/55	0.26
Lymphovascular invasion (%)^b^	18 (25)	38 (69)	< 0.0001
Concomitant subtype histology (%)^b^	13 (18)	21 (38)	0.016
Concomitant CIS (%)^b^	10 (14)	16 (29)	0.047
Pathological LN metastasis, positive/negative/no LN dissection^b^	2/16/53	8/17/30	0.021
Positive surgical margin (%)^b^	4 (6)	10 (18)	0.043
High budding (%)^b^	4 (6)	37 (67)	< 0.0001
High TAICs (score 2–3) (%)^b^	21 (30)	29 (53)	0.010

*CIS* Carcinoma in situ, *LN* Lymph node, *TAICs* Tumor-associated immune cell status

^a^ Student’s *t*-test, ^b^ Fisher’s exact test

### Overall and cancer-specific survival

**Fig. 3:**
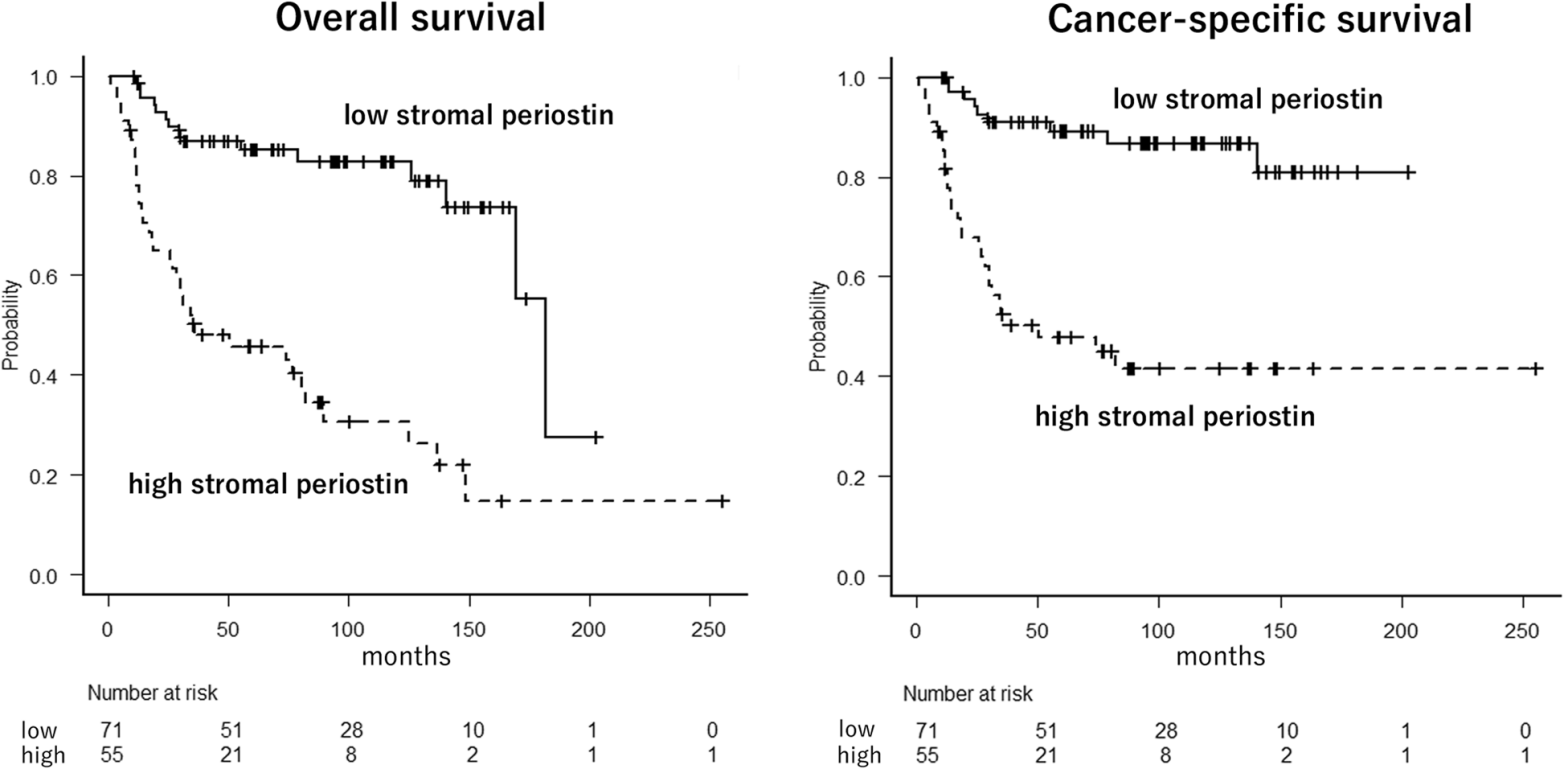
Impact of stromal periostin expression on overall survival and cancer-specific survival in upper urinary tract urothelial carcinoma. Low stromal periostin expression (bold line) vs. high stromal periostin expression (dashed line): overall and cancer-specific survival, each *p* < 0.0001

The median follow-up time was 60 months (range: 1–254 months). The 5-year overall survival and cancer-specific survival rates were 47% and 51%, respectively, in patients with high stromal periostin expression, and 86% and 90%, respectively, in patients with low stromal periostin expression. The overall and cancer-specific survival curves for the 126 patients with UTUC stratified by stromal periostin expression are presented in Fig. 3. High stromal periostin expression was significantly associated with significantly shorter overall and cancer-specific survival based on the log-rank test (*p* < 0.0001, each).

Cox univariate analysis showed that pT category ≥ 3, LVI, pathological lymph node metastasis (positive vs. negative/no lymph node dissection), positive surgical margins, high budding, and high stromal periostin expression were correlated with lower overall survival (Table 2a). Cox multivariate analysis, including these six variables revealed that high stromal periostin expression was an independent predictor of overall survival (Table 2b; *p* = 0.00072, hazard ratio = 3.62).

**Table 2:** Cox regression model estimates of the significance of predictive factors for overall survival

Variables	*p v*alue	HR (95% CI)
(a) Univariate Cox regression model		
Non-papillary gross finding	0.062	1.76 (0.97–3.18)
Pathological T category ≥ 2	0.086	1.94 (0.91–4.12)
Pathological T category ≥ 3	0.018	2.03 (1.13–3.63)
High grade tumor	0.87	1.17 (0.16–8.54)
Lymphovascular invasion	< 0.0001	3.27 (1.84–5.80)
Concomitant subtype histology	0.097	1.68 (0.91–3.09)
Concomitant carcinoma in situ	0.23	0.61 (0.28–1.37)
Pathological lymph node metastasis^a^	0.043	2.34 (0.99–5.54)
Positive surgical margin	0.020	2.39 (1.15–4.96)
High budding	< 0.0001	3.82 (2.19–6.67)
High TAICs (score 2–3)	0.51	1.20 (0.69–2.09)
High periostin expression	< 0.0001	4.92 (2.68–9.01)
(b) Multivariate Cox regression model		
Pathological T category ≥ 3	0.42	0.74 (0.35–1.55)
Lymphovascular invasion	0.074	1.97 (0.94–4.13)
Pathological lymph node metastasis^a^	0.73	0.82 (0.27–2.47)
Positive surgical margin	0.31	1.59 (0.65–3.89)
High budding	0.43	1.38 (0.62–3.04)
High periostin expression	0.00072	3.62 (1.72–7.64)

*CI* Confidence interval, *HR* Hazard ratio, *TAICs* Tumor-associated immune cell status

^a^ Pathologically positive nodes vs. pathologically negative nodes/no lymph node dissection

Considering cancer-specific survival, Cox univariate analysis indicated that a pT category ≥ 3, LVI, concomitant subtype histology, pathological lymph node metastasis, high budding, and high stromal periostin expression were poor prognostic predictors (Table 3a). Subsequent Cox multivariate analysis showed that LVI (*p* = 0.032, hazard ratio = 2.61) and high stromal periostin expression (*p* = 0.020, hazard ratio = 3.07) were independent predictors of cancer-specific survival (Table 3b).

**Table 3:** Cox regression model estimates of the significance of predictive factors for cancer-specific survival

Variables	*p* value	HR (95% CI)
(a) Univariate Cox regression model		
Non-papillary gross finding	0.076	1.82 (0.94–3.53)
Pathological T category ≥ 2	0.084	2.29 (0.89–5.87)
Pathological T category ≥ 3	0.012	2.54 (1.23–5.24)
High grade tumor	0.95	1.12 (0.08–5.45)
Lymphovascular invasion	< 0.0001	5.05 (2.43–10.50)
Concomitant subtype histology	0.026	2.12 (1.10–4.12)
Concomitant carcinoma in situ	0.61	0.81 (0.35–1.83)
Pathological lymph node metastasis^a^	0.019	2.85 (1.19–6.84)
Positive surgical margin	0.063	2.17 (0.96–4.98)
High budding	< 0.0001	6.15 (3.12–12.1)
High TAICs (score 0–1)	0.18	1.54 (0.82–2.91)
High periostin expression	< 0.0001	5.90 (2.79–12.52)
(b) Multivariate Cox regression model		
Pathological T category ≥ 3	0.30	0.61 (0.24–1.54)
Lymphovascular invasion	0.032	2.61 (1.08–6.27)
Concomitant subtype histology	0.084	1.85 (0.92–3.72)
Pathological lymph node metastasis^a^	0.91	1.05 (0.41–2.69)
High budding	0.079	2.26 (0.91–5.63)
High periostin expression	0.020	3.07 (1.19–7.88)

*CI* Confidence interval, *HR* Hazard ratio, *TAICs* Tumor-associated immune cell status

^a^ Pathologically positive nodes vs. pathologically negative nodes/no lymph node dissection

We performed Cox univariate analysis for eight tumors with focal/weak epithelial periostin expression. No statistically significant correlation was observed between these tumors and lower overall survival (*p* = 0.090, hazard ratio = 2.23) or cancer-specific survival (*p* = 0.11, hazard ratio = 2.33).

## Discussion

Risk stratification for managing UTUC based on postoperative specimens is of utmost importance, particularly due to technical and interpretational challenges with cytohistopathological screening, including voided/washing cytology and flexible ureteroscopic biopsy [2, 28]. In the present study, high stromal periostin expression was associated with non-papillary gross findings, higher pT category, LVI, concomitant carcinoma in situ, subtype histology, lymph node metastasis, positive surgical margins, high tumor budding, and high TAICs. Multivariate Cox analysis revealed that high stromal periostin overexpression was an independent predictor of overall survival, and LVI and stromal periostin overexpression were independent predictors of cancer-specific survival. In contrast, only weak and focal epithelial periostin expression was detected in a small subset of tumors; no UTUC cases were considered to have high epithelial periostin expression.

Upregulation of periostin is associated with adverse clinicopathological factors and poor patient outcomes in genitourinary cancers such as prostate, renal, and penile cancers [29]. In contrast, the results of previous studies in search of periostin’s role in urinary bladder cancer are controversial [19, 20, 30–32]. Kim et al. reported that downregulation of periostin mRNA expression was observed in three selected human bladder cell lines and that ectopic periostin expression in SBT31A bladder cancer cells suppressed in vitro cellular invasiveness [19]. They also demonstrated that inhibition of Akt by periostin induced the upregulation of E-cadherin and suppressed the invasiveness of bladder cancer cells [30]. Conversely, Silvers et al. performed quantitative real-time polymerase chain reaction in 10 human bladder cell lines and observed high periostin expression in high-grade bladder cancer cell lines J82, TCC-SUP, and UMUC3 [20]. This contradiction with the data from Kim et al. was explained as resulting from possible undefined, cell line-specific functions of different periostin splicing variants that were also reported in the specific bladder cell lines used [20, 31]. In their study, immunohistochemical analysis using tissue microarray revealed that periostin reactivity in muscle-invasive bladder cancer cells (i.e., epithelial expression) was correlated with worse patient prognosis, and there was no significant correlation between epithelial periostin expression and the recurrence of non-muscle-invasive bladder cancers [20], consistent with another study [32]. However, no study has provided information on stromal periostin expression in bladder cancer. In contrast, the present study indicated that only weak and focal epithelial periostin expression was detected in UTUCs and was not correlated with poor prognosis, whereas high stromal periostin expression was observed in 44% of cases with a significant prognostic impact. Despite differences in immunohistochemistry protocols and criteria defining periostin overexpression in the setting of the same primary antibody among studies (rabbit polyclonal antibody, Ab14041), this discrepancy may also arise from molecular genetic differences between bladder urothelial carcinoma and UTUC, as recent studies indicate [33, 34]. Interestingly, the molecular features of UTUC include more frequent “luminal” and “luminal infiltrative” subtypes, characterized by a strong stromal signature, as compared to bladder cancer [33, 34]. Using 20 transurethral resection specimens of bladder urothelial carcinomas, we provisionally analyzed periostin expression by immunohistochemistry and “moderate” but focal epithelial expression was detected in 2 cases (data not shown). Additionally, it is worth of further investigation using other techniques, such as mRNA in situ hybridization and/or reverse transcription-polymerase chain reaction, to clarify its cellular function.

In the present study, high stromal periostin expression was significantly associated with high budding in UTUCs. Several studies have also indicated a close relationship between stromal periostin overexpression and histological small clustering and/or isolated invasive patterns of cancer cells at the tumor front, such as tumor budding in colorectal adenocarcinoma [35] and “pattern of invasion” in oral squamous cell carcinoma [36]. Overexpression of periostin co-localizes with fibronectin and collagen, thereby promoting an extracellular matrix organization through the activation of focal adhesion kinase and PI3K/Akt signaling [13]. The activated stroma corresponds to a high-grade epithelial-mesenchymal transition, possibly represented by histological small clusters/isolated invasive patterns of cancer cells (i.e., tumor budding), and promotes cancer invasion and metastasis [13, 20, 37]. With regard to the prognostic power, the current study demonstrated that the status of stromal periostin expression stratified patients by survival outcome more effectively than the status of tumor budding, consistent with a previous study [36]. Although tumor budding can be assessed only on H&E-stained slides, it has been indicated that accurate and reproducible determination has been difficult, particularly in terms of the identification of single cancer cells and small cell clusters [38]. In addition, some anatomical characteristics of the upper urinary tract, such as renal tubules and collecting ducts in the renal parenchyma, occasionally obscure invasive cancer cells [7], whereas the peritumoral stroma is insusceptible to this matter. Although further investigation is necessary, high stromal periostin expression on immunohistochemistry can be a useful prognostic marker for UTUCs.

Emerging evidence has demonstrated that periostin plays a key role in chronic inflammation of several non-neoplastic and neoplastic lesions [18]. Serum periostin is an established biomarker of Th2 driven immunoreaction in asthma and allergic dermatitis [18, 39]. In breast cancer and skin melanoma, stromal periostin expression is significantly associated with the number of infiltrated M2 macrophages, which are essential components of the tumor immune microenvironment involved in tumor progression and metastasis [17, 40]. The present study also indicated a significant relationship between high stromal periostin expression and high TAICs in examined UTUCs, whereas TAICs was not correlated with patient outcomes. Although previous studies on TAICs and/or tumor-infiltrating lymphocytes (TILs) in urothelial carcinoma have mainly focused on urinary bladder cancer, a few studies have analyzed the tumor immune microenvironment of UTUC and presented different results. Two studies reported that high stromal TILs can predict improved survival [41, 42]. Conversely, Nukui et al. found that low stromal TILs combined with low epithelial programmed cell death-1 ligand 1 expression predicted increased survival [43]. The variations in the results might arise from the inconsistent analytical methods, small sample size, heterogeneity of urothelial carcinoma, and complexity of the underlying immune regulatory pathway. High TAICs/TILs, which correlate with high tumor burden, have also been recognized as predictive markers of immune checkpoint inhibitors, and detailed investigation of the tumor immune microenvironment in a larger cohort of UTUCs would help improve therapeutic strategies and prognosis prediction.

Our study had several limitations. First, because this was a retrospective study with a relatively small sample size, investigations subdivided by tumor location (renal pelvic and ureteral tumors) could not be performed. Second, although whole slide immunohistochemical analysis rather than tissue microarray analysis was performed, interobserver bias could not be completely excluded. To reduce bias, two pathologists separately evaluated periostin expression status, and there were no cases in which the judgment of the two pathologists in terms of periostin expression status differed. Finally, because there was a subset of cases that did not undergo lymph node dissection, the actual number of cases with “pathological lymph node metastasis” might be higher than the present data; consequently, pathological lymph node metastasis may be underestimated as a clinicopathological variable.

## Conclusions

We demonstrated that high stromal periostin expression, but not epithelial expression, was often observed in invasive UTUCs examined and correlated with several adverse clinicopathological factors. Despite the need for immunohistochemistry, stromal periostin expression has been suggested to have a greater prognostic impact than other histological factors, including tumor budding and TAICs. Its molecular function and relevance in the clinical management of UTUC should be explored in future studies.

## Data Availability

The datasets used and/or analyzed during this study are available from the corresponding author upon reasonable request.

## Abbreviations

H&E: Hematoxylin and eosin
LVI: Lymphovascular invasion
PI3K/Akt: Phosphatidylinositol-3-kinase/protein kinase B
pT: Pathological T
TAICs: Tumor-associated immune cells
Th2: T helper 2
TILs: Tumor-infiltrating lymphocytes
UTUC: Upper urinary tract urothelial carcinoma
WHO: World Health Organization
